# Higher vs. Lower DP for Ventilated Patients with Acute Respiratory Distress Syndrome: A Systematic Review and Meta-Analysis

**DOI:** 10.1155/2019/4654705

**Published:** 2019-07-18

**Authors:** Zhen Chen, Xuxia Wei, Genglong Liu, Qiang Tai, Donghua Zheng, Wenfeng Xie, Li Chen, Ganping Wang, Jia-Qi Sun, Siqi Wang, Na Liu, Haijin Lv, Liuer Zuo

**Affiliations:** ^1^Intensive Care Unit, Shunde Hospital, Southern Medical University (The First People's Hospital of Shunde), Foshan 528308, Guangdong Province, China; ^2^Surgical Intensive Care Unit, The Third Affiliated Hospital of Sun Yat-sen University, Guangzhou 510630, Guangdong Province, China; ^3^Department of Pathology, Affiliated Cancer Hospital & Institute of Guangzhou Medical University, Guangzhou, 510095, Guangdong Province, China; ^4^Intensive Care Unit, The First Affiliated Hospital, Sun Yat-sen University, Guangzhou, 510080, Guangdong Province, China

## Abstract

**Objectives:**

Driving pressure (DP) has recently become a promising mediator for the identification of the effects of mechanical ventilation on outcomes in acute respiratory distress syndrome (ARDS). The aim of this study was to systematically and quantitatively update and assess the association between DP and mortality among ventilated patients with ARDS.

**Methods:**

PubMed, the Cochrane Library, ISI Web of Knowledge, and Embase were systematically searched from inception to June 2018. Two investigators conducted the literature search study selection, data extraction, and quality evaluation independently. RevMan 5.3 software was used for all statistical analyses.

**Results:**

A total of seven studies comprising 8010 patients were included in this meta-analysis. Higher DP showed a significant association with higher mortality (pooled risk ratio, 1.10; 95% [CI], 1.05–1.16;* I*^2^ =58%). Sensitivity analysis indicated that one study significantly affected the stability of pooled results. One of the subgroups investigated, ARDS severity, could account for the heterogeneity. An exploratory post hoc subgroup analysis and higher DP significantly increased mortality in the mild to severe ARDS subgroup (RR 1.28; 95% [CI], 1.14–1.43; ***I***^2^ =0), but not in the moderate to severe ARDS subgroup (RR 1.18; 95% [CI], 0.95–1.46;  ***I***^2^ =52%).

**Conclusion:**

Higher DP was significantly associated with an increased risk of death among ventilated patients with ARDS. But it did not seem to predict prognosis to moderate to severe ARDS. Future prospective randomized clinical trials are needed to verify the results of this meta-analysis and address the unresolved questions about optimum cutoff values for DP.

**Trial Registration:**

This trial is registered with PROSPERO (CRD42018102146), on 11 August 2018.

## 1. Background

Acute respiratory distress syndrome (ARDS) is a common disease that affects up to 20% of mechanically ventilated patients during an intensive care unit (ICU) stay [1]. Despite decades of research, seldom effective therapeutic strategies for treating clinical ARDS have appeared. Current treatments focus on support, as mechanical ventilation is a cornerstone life-saving treatment for ARDS. Lung protective ventilation acts by limiting the iatrogenic injury that is linked to mechanical ventilation [2, 3] and is frequently used ventilation method. It includes several components, the most important of which is lowering tidal volume (V_T_), limiting plateau (Pplat) to or below 30 cmH_2_O and higher positive end-expiratory pressures (PEEPs). This combined strategy is indeed the valid ventilator intervention that has been indicated to prominently improve survival to date [4, 5].

To optimize lung protective ventilation, a host of studies have introduced the range of V_T_ to predict body weight (PBW) to normalize V_T_ to lung size [6, 7]. In ARDS, due to the presence of lung disease, there is a commonly significant and nonuniform reduction in the amount of lung available for ventilation among patients [8]. Therefore, a similar tidal volume (VT) dependent on ideal body weight can vary for different degrees of lung stress [9]. In contrast, driving pressure (DP), which is calculated as end-inspiratory plateau pressure (Pplat) minus applied positive end-expiratory pressure (PEEP) and is equivalent to the ratio between the V_T_ and compliance of the respiratory system, can better reflect lung injury compared to V_T_ adjustments based solely on ideal body weight. [10]

A retrospective analysis of several trials in patients with ARDS comparing different PEEP levels at the same V_T_ or different V_T_ levels at the same PEEP, or a combination of both, found that DP was more strongly related to mortality than was Pplat [11]. Similarly, a recent meta-analysis demonstrated an association between DP and mortality [12]. Nevertheless, a few new studies [13, 14] offer additional data that can provide a clearer understanding of the potential value of DP for ARDS. We executed an updated systematic review and meta-analysis to add further documentation that confirms the association of DP with mortality in mechanically ventilated patients with ARDS.

## 2. Methods

The present meta-analysis was reported according to the Preferred Reporting Items for Systematic Reviews and Meta-analyses Statement (PRISMA) [15] (Supplementary Appendix 1). The review protocol was registered at the PROSPERO registry of systematic reviews in August 2018 (registry number: CRD 42018102146).

### 2.1. Data Sources

We systematically searched four databases, PubMed, the Cochrane Library, ISI Web of Knowledge, and Embase, from inception to June 2018, using a sensitive search strategy (Supplementary Appendix 2). A basic search was performed using the following vocabulary terms (when available), text words, and keywords: (“driving pressure [with related synonyms]”) AND (“acute respiratory distress syndrome [with related synonyms]” OR “ARDS [with related synonyms]”). No language restriction was applied for article selection. Additional studies were identified by reviewing the reference lists of relevant articles.

### 2.2. Eligibility Criteria

Two reviewers (LEZ and ZC) independently evaluated the resulting studies for their eligibility for inclusion. In cases of disagreement, a consensus was reached by discussion or by consultation with a third reviewer (HJL). Randomized controlled trials (RCTs), controlled studies, cohort studies, secondary analysis studies, and case-control studies were considered eligible if they collected data on mortality in ventilated adult patients with ARDS with DP measurement. The exclusion criteria were as follows: editorials, reviews, abstracts or conference proceedings, expert opinions, animal experiments, unrelated intervention or outcomes, and insufficient information to extract data after contacting the corresponding authors.

### 2.3. Data Extraction

Two reviewers (GLL and XXW) independently extracted study characteristics and data from each eligible study, including the authors, year of study, country of origin, study design, study settings, relevant population, sample size, mean age, the optimal cutoff values, outcome assessment, and follow-up period. The diagnosis of ARDS met the Berlin criteria [16] or was based on the American-European consensus definition criteria [17]. Our primary outcome was hospital mortality; if not available, we used mortality at the latest reported time point. If a meta-analysis noted that unpublished data were provided by the primary authors, we extracted those data from forest plots of the meta-analysis [12] and reviewed original articles to confirm whether the trials met our inclusion criteria. When those data were our outcomes of interest, we pooled them with the data from primary trials. Additionally, a study by Villar et al. [18] contained two data sets (derivation cohort and validation cohort). The authors have shared the corresponding data, which can be downloaded from http://links.lww.com/CCM/C436.

### 2.4. Quality Assessment

The Newcastle Ottawa Scale for cohort studies was used to assess the reporting quality of the included component studies [19]. This scale comprises eight items evaluating the quality of observational cohort studies in terms of selection, comparability, and outcome. Observational cohort studies receiving seven or more stars were considered to be of high quality. The assessment was performed independently by two reviewers (XXW and ZC). Disagreements were resolved by consensus.

### 2.5. Statistical Analyses

RevMan 5.3 software from the Cochrane Collaboration was utilized for the meta-analysis. Relative risk (RR) was used as the common measure of association across studies. To this end, the hazard ratios (HRs) were directly considered the RRs. In addition, an adjusted RR or HR was selected as the effect size from eligible studies based on multivariate analysis. RR was reported to estimate the association between DP and mortality among ventilated patients with ARDS. RR and the associated 95% CI were pooled using fixed-effect (Mantel-Haenszel method) or random-effect models (DerSimonian and Laird method) [20]. RRs greater than 1 indicated a beneficial effect of the exposure for mortality.

Heterogeneity in a meta-analysis indicates the degree of variability in the results across studies and was appraised using the Q test, p value, and I^2^ index, which included thresholds for low (I^2^ < 50%), moderate (50% < I^2^ <75%), and high (I^2^ >75%) heterogeneity [21].

In addition, to investigate the potential sources of heterogeneity in the eligible studies, sensitivity analyses were performed in RevMan with sequential exclusion of each study to explore the heterogeneity observed. Sensitivity analysis was also investigated by removing trials with characteristics different from the others.

To evaluate whether the association between higher DP and mortality among ventilated patients with ARDS was modified by clinical characteristics, several subgroups were examined based on ARDS severity (mild to severe ARDS vs. moderate to severe ARDS), sample size (>500 vs.≤500), and cutoff value (>15 vs.≤15). On account of a trial by Chiu et al. [13] employing ECOM as the main cotreatment and a study by Raymondos et al. [14] missing important parameter (cutoff value of DP), we will exclude the above two trials to perform an exploratory post hoc subgroup analysis based on ARDS severity (mild to severe ARDS vs. moderate to severe ARDS). Analysis was performed to assess whether the difference between the subgroups was statistically significant. We used *χ*2 to test for subgroup differences—that is, whether the observed differences in the subgroups are compatible with chance alone. A low ***P*** value (or a large *χ*2 statistic relative to its degree of freedom) provides evidence of heterogeneity beyond chance.

Funnel plots were used to screen for potential publication bias. We calculated *κ* statistics to assess the agreement between the two investigators for assessment of methodological quality.

## 3. Results

### 3.1. Identification of Studies

**Figure 1:**
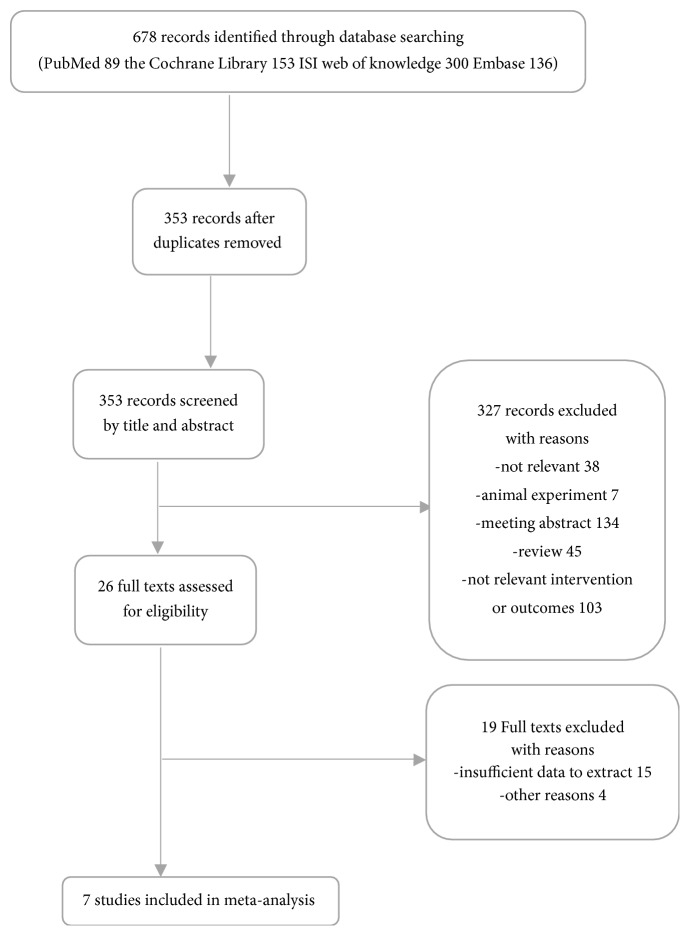
Flow diagram of literature search and selection process of the studies.

The flow chart of the study selection procedure is shown in Figure 1. The initial search identified 89 citations from PubMed, 153 from the Cochrane Library, 300 from ISI Web of Knowledge, and 136 from Embase. After removing 325 duplicates, the titles and abstracts of the remaining 353 papers were screened. After 326 records were eliminated by inspection of the titles and abstracts, 26 articles were subsequently scrutinized by a reading of the full text. As a result, 7 studies [11, 13, 14, 18, 22–24] fulfilled our eligibility criteria and were included in the final meta-analysis.

### 3.2. Characteristics of the Included Studies

The characteristics of the eligible studies are presented in Table 1. Two secondary analyses of previous RCTs [11, 22], one secondary analysis of previous cohorts [18], one retrospective observational study (ROS) [13], and three prospective observational studies (POS) [14, 23, 24] were included, all [11, 13, 14, 18, 22–24] of which were published between 2015 and 2017. With respect to clinical setting, all seven studies [11, 13, 14, 18, 22–24] were conducted in an intensive care unit (ICU). The sample sizes varied across the studies, ranging from 150 to 3562, and the mean age of the patients was between 50.3 and 62.8 years. In terms of population, four studies [11, 14, 23, 24] focused on mild to severe ARDS patients, and three studies [13, 18, 22] focused on moderate to severe ARDS patients. With regard to criteria for selecting the DP thresholds, each study provided optimum cutoff points. The cutoff values of DP for the prediction varied across the studies, ranging from 13 to 21 cmH_2_O, with the exception of the study by Raymondos et al. [14], in which the cutoff point was not reported.

### 3.3. Results of the Quality Assessment

The interrater reliability for the assessment of quality items was 0.73 (***P***<0.0001). Overall, the methodological quality was moderate. Details of the methodological assessment are shown in Table 2. Table 2 displays the quality assessment using the Newcastle Ottawa Scale for observational studies. The results showed that three studies scored 9 points, and the remaining studies scored 8 points. That is, the included studies showed a low risk of bias.

### 3.4. Data Synthesis

**Figure 2:**
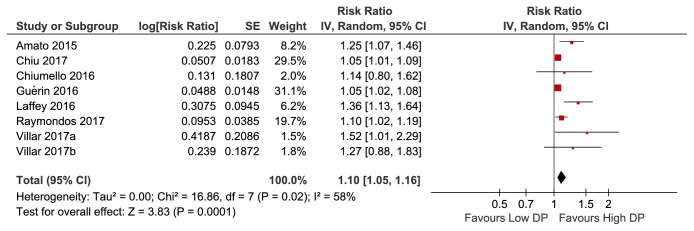
Forest plots for pooled risk ratio of high DP versus low DP from eligible studies.

**Figure 3:**
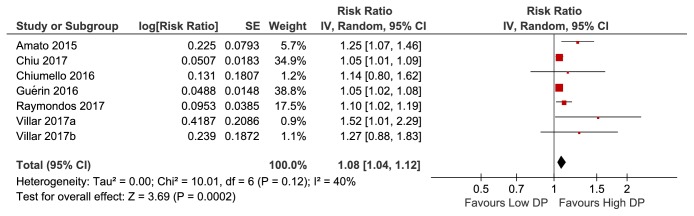
Sensitivity analysis by excluding study by Laffey et al.

In the meta-analysis of eight studies involving 8010 patients, higher DP was significantly associated with increased mortality among mechanically ventilated ARDS patients (pooled risk ratio, 1.10; 95% [CI], 1.05–1.16;* I*^2^ =58%) (Figure 2). Considering the remarkable heterogeneity across studies observed, a sensitivity analysis was performed to explore the heterogeneity. After omitting one study by Laffey et al. [24], the heterogeneity of the pooled RR (1.08; 95% [CI], 1.04–1.12;* I*^2^ =40%) showed a relative decrease from moderate to low heterogeneity (Figure 3), with the* I*^2^ index decreasing from 58% to 40%. Given a study by Chiu et al. [13] with ECMO as the main cointervention, a sensitivity analysis in which the trials by Chiu et al. were excluded showed a pooled RR of 1.16 (95% [CI], 1.07–1.26;* I*^2^ =63%) (Supplementary Figure S1). On account of a study by Raymondos et al. missing important parameter (cutoff value of DP), a sensitivity analysis in which the trials by Raymondos et al. [14] were excluded showed a pooled RR of 1.11 (95% [CI], 1.05–1.18;* I*^2^ =63%) (Supplementary Figure S2).

**Figure 4:**
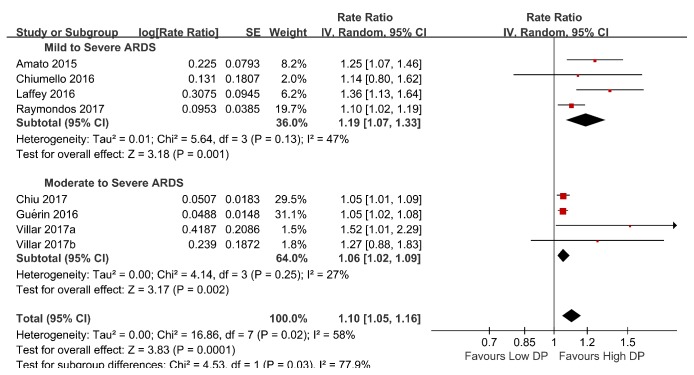
Subgroup analysis-ARDS severity for the predictive value of elevated DP for mortality in ARDS with mechanical ventilation.

**Figure 5:**
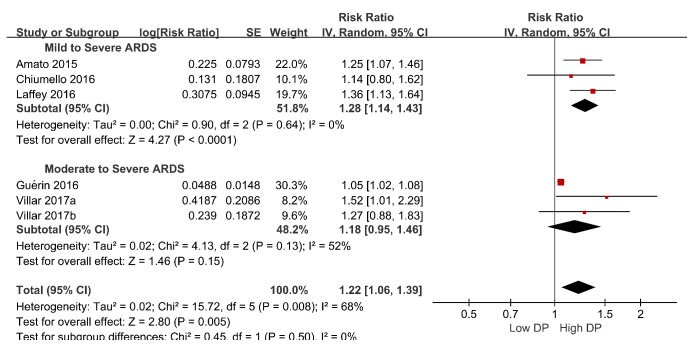
An exploratory post hoc subgroup analysis based on ARDS severity.

**Figure 6:**
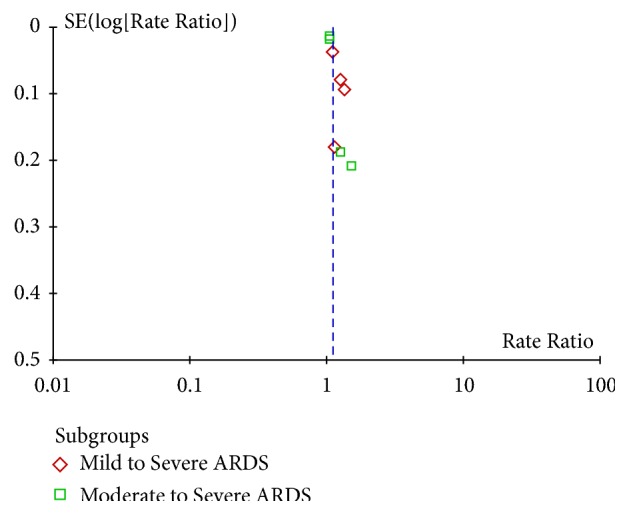
Funnel plot of publication bias.

Additionally, subgroup analyses were performed based on ARDS severity, sample size, and cutoff values. One of the subgroups investigated, ARDS severity, could account for the heterogeneity. In the mild to severe ARDS subgroup, the pooled RR was 1.19 (95% CI, 1.07–1.33;* I*^2^ =47%). In the moderate to severe ARDS subgroup, the pooled RR was 1.06 (95% CI, 1.02–1.09;* I*^2^ =20%) (Figure 4). The relative risk was higher in the mild to severe ARDS subgroup than the moderate to severe ARDS subgroup (*χ*2= 4.53, **P** = 0.03). The results of all subgroup analyses are presented in Table 3. An exploratory post hoc subgroup analysis based on ARDS severity was performed. In the mild to severe ARDS subgroup, the pooled RR was 1.28 (95% CI, 1.14–1.43;* I*^2^ =0). In the moderate to severe ARDS subgroup, the pooled RR was 1.18 (95% CI, 0.95–1.46;* I*^2^ =52%) (Figure 5). Inspection of the corresponding funnel plot revealed no evidence of significant publication bias (Figure 6).

## 4. Discussion

The present systematic review and meta-analysis investigated the significant association of DP with mortality among ventilated patients with ARDS. Accordingly, the pooled risk ratio is 1.10 (95% CI, 1.05–1.16), indicating that higher DP is a bedside-available parameter for the prediction of mortality in ventilated patients with ARDS that may help identify patients who are at increased risk of death. An exploratory post hoc subgroup analysis indicated it seems to have no prognostic effect on moderate to severe ARDS.

A previous meta-analysis reported a similar topic [12]. The differences between the present meta-analysis and the previous one are as follows. First, our meta-analysis included two additional trials providing sufficient information for analysis that were not included in previous meta-analyses. As the latest and most comprehensively updated meta-analysis, the present study further reinforces the results of previous meta-analyses. Second, we registered the protocol of this study on PROSPERO. A registered protocol may increase the transparency and quality of meta-analysis.

Our meta-analysis confirmed that the higher DP significantly increased mortality among ventilated patients with ARDS, in accord with result of published meta-analyses [12]. Significant heterogeneity was observed in the present study. We conducted a sensitivity analysis with serial exclusion of individual studies. After omitting one study by Laffey et al. [24], it could account for the heterogeneity. A possible explanation is that the trial did not primitively report the relative risk for higher DP and mortality in entire ARDS populations. We extract the effect size from a published meta-analysis [12], which may affect the accuracy. To investigate other possible reasons for study heterogeneity, we performed subgroup analysis. A subgroup analysis based on ARDS severity indicated DP was consistently associated with increased mortality with low heterogeneity in both subgroups. However, analysis of subgroup differences found the relative risk was significantly higher in the mild to severe ARDS subgroup than the moderate to severe ARDS subgroup (***P*** = 0.03). Given two trials by Chiu et al. [13] and Raymondos et al. [14] including their own limitations, we removed the above two trials to perform an exploratory post hoc subgroup analysis based on ARDS severity. The result showed that higher DP is still related to increased mortality in the mild to severe ARDS subgroup, but not in the moderate to severe ARDS subgroup. Higher DP appears to have no prognostic effect on moderate to severe ARDS. Future prospective randomized clinical trials are needed to verify the results of this meta-analysis.

Likewise, the importance of DP in determining the effects of ventilator settings has been subsequently confirmed by a recent epidemiological study involving more than 2000 patients with ARDS in 50 countries [25]. Higher survival was detected in patients with DP≤14 cmH_2_O at the onset of the syndrome. Furthermore, in a recent secondary analysis study, [26] DP showed an independent association with mortality [adjusted OR, 1.04 (95% CI, 1.01 - 1.07)] among mechanically ventilated patients without ARDS. DP may be the most useful ventilator variable for stratifying patients' disease severity and the risk of ventilator-induced lung injury (VILI) among mechanically ventilated patients, whether or not the patients suffer from ARDS.

To date, several ventilator variables, such as PEEP and V_T_, have been determined and monitored for their relative effects on survival among ventilated patients with ARDS. In a previous systematic review and meta-analysis, [27, 28] PEEP or V_T_ was not related to increased mortality in ARDS patients receiving lung protective ventilation. Nevertheless, the current study confirms the results of prior individual studies indicating the association of higher DP with higher mortality. Gattinoni et al. [29] recently obtained deep insight into the importance of the primary components of lung-protective mechanical ventilation. The authors calculated the mechanical power applied to respiratory system based on the assumption that the greater the power is, the greater the likelihood of lung injury becomes. The main variables associated with the ventilator, including V_T_, respiratory rate, flow rate, PEEP, DP, and patient compliance and airway resistance, all exert an influence on lung injury. The strongest effects are due to V_T_ and DP, with the weakest effect derived from the application of PEEP. Hence, all these parameters must be considered when predicting outcomes. The diverse etiologies and varying severities of ARDS imply that prediction of the risk of death is impossible to determine by a single parameter. Apparently, all ARDS patients should be ventilated with a lung-protective Vt, PEEP, Pplat, and DP.

Currently, no study has prospectively evaluated whether systematic interventions titrated to DP reduction may provide a relevant clinical benefit. Thus, a well-performed RCT that evaluates outcomes from VT adjustments based on DP compared to VT adjustments based solely on ideal body weight must be performed to ascertain the benefit of using DP to set volume. Moreover, a safety and feasibility trial is also needed to understand how a protocol targeting DP can be developed and implemented by bedside clinicians (e.g., adjustment of tidal volume or other ventilatory variables, lung recruitment with PEEP) and whether there are any adverse effects of such a strategy compared to convention mechanical ventilation strategies.

Several limitations of this study should be discussed. First, marked heterogeneity existed across the included studies in terms of ARDS severity, sample size, and optimal cutoff values. Although we performed sensitivity and subgroup analyses to explore the sources of potential heterogeneity between studies, the heterogeneity of each parameter was not entirely reduced. Additional high-quality studies with well-designed may be required. Second, the cutoff points of DP varied, ranging from 13 to 21 cmH_2_O, and we could not determine ideal cutoff values for DP because we did not have the raw data to construct ROC curves. To confirm whether one or more DP thresholds exist, further explorations in larger, prespecified groups of patients are required. Third, one study by Amato et al. [11] is an analysis of several prior trials, and another study by Guerin et al. [22] is an analysis of two prior trials. An individual patient data (IPD) meta-analysis should be appropriately performed to account for the random effects of the studies whose data involved 2-3 trials. However, we do not have access to the data underlying the original studies. Finally, we could not determine whether the data rooted in each trial, namely, the numerical value of the DP, are contaminated by several confounding factors that strongly affect results (e.g., spontaneous effort, chest wall stiffness, and position). [30] Simultaneously, virtually none of the trials ensured that DP was recorded under passive conditions because plateau pressure can be displayed by most ventilators even when the patient is actively breathing. Besides, DP cannot be recorded when there is reverse triggering occurring during inspiration [31, 32]. Again, chest wall stiffness and, in some cases, position affect those values as well. DP will change in the same patient with variation of disease and ventilation settings. Therefore, the predictive accuracy of DP needs to be further studied to evaluate.

## 5. Conclusion

Higher DP was significantly associated with an increased risk of death among ventilated patients with ARDS. Yet it did not appear to predict prognosis to moderate to severe ARDS. Future prospective randomized clinical trials are needed to verify the results of this meta-analysis and address unresolved questions about the optimum cutoff values of DP.

## Figures and Tables

**Table 1:** Characteristics of the studies included in the meta-analysis.

Study	Study design	Country	Setting	Population	Sample Size	Male (%)	Age (years)	Mortality (%)	Cut-off value (cmH_2_0)	Follow-up (days)
Amato et al. 2015	ROS	Brazil	ICU	Mild to Severe ARDS	3562	NR	NR	34.7	15	Mortality at day 60
Chiu et al. 2017	ROS	Taiwan	ICU	Severe ARDS with ECMO	158	68.4	50.3	55.1	21	ICU mortality
Chiumello et al. 2016	POS	Italy	ICU	Mild to Severe ARDS	150	68	62	32	15	ICU mortality
Guerin et al. 2016	POS	France	ICU	Moderate to severe ARDS	787	68.9	59	32.3	13	Mortality at day 90
Laffey et al . 2016	POS	Canada	ICU	Mild to severe ARDS	2377	61.9	60.5	40.1	14	Hospital mortality
Raymondos et al. 2017	POS	Germany	ICU	Mild to Severe ARDS	198	71.7	59/62.8	57.5	NR	Hospital mortality
Villar et al. 2017a	ROS	Spain	ICU	Moderate to Severe ARDS	478	69.9	54	42.2	19	Hospital mortality
Villar et al. 2017b	ROS	Spain	ICU	Moderate to Severe ARDS	300	67	57	42.3	19	Hospital mortality

ICU=intensive care unit, ARDS=acute respiratory distress syndrome, NR=not reported, ROS= retrospective observational study, ECMO=extracorporeal membrane oxygenation, POS= prospective observational study.

**Table 2:** Quality Assessment with Newcastle Ottawa Scale for cohort study.

Study	Selection				Comparability		Outcome		
	REC	SNC	AE	AOI	Design and Analysis	Assessment	Enough Follow-up	Adequate Follow-up	Score
Amato et al. 2015	☆	☆	☆	☆	☆☆	☆	☆	☆	9
Chiu et al. 2017	☆	☆	☆	☆	☆☆	☆	☆	☆	9
Chiumello et al. 2016	☆	☆	☆	☆	☆☆	☆	☆		8
Laffey et al. 2016	☆	☆	☆	☆	☆☆	☆	☆	☆	9
Raymondos et al. 2017	☆	☆	☆	☆	☆	☆	☆	☆	8
Villar et al. 2017	☆	☆	☆	☆	☆☆	☆	☆		8
Guerin et al. 2016	☆	☆	☆	☆	☆	☆	☆	☆	8

REC=Representative of Exposed Cohort, SNC=Selection of Nonexposed Cohort, AE=Ascertainment of Exposed, AOI=Absence of Outcome of Interest, star (☆) was allocated to a particular item when it was adequately reported and addressed. The item “comparability” could be allocated with a maximum of two stars. Dashes indicate this item was not adequately reported or addressed.

**Table 3:** Results of subgroup analysis based on different standards.

	K	N	RR [95% CI]	*P*	Study heterogeneity				P (between-groupcomparison)
					Chi^2^	*df*	I^2^ (%)	P	
ARDS Severity									0.03
Mild to Severe ARDS	4	6287	1.19 [1.07, 1.33]	0.001	5.64	3	47	0.13	
Moderate to Severe ARDS	4	1723	1.06 [1.02, 1.09]	0.002	4.14	3	27	0.25	
Sample size									0.28
Sample Size≤500	5	1284	1.08 [1.02, 1.14]	0.005	5.09	4	21	0.28	
500<Sample Size	3	6726	1.19 [1.00, 1.42]	0.05	11.76	2	83	0.003	
Cut-off value									0.97
DP≤15cmH_2_O	4	6876	1.18 [1.02, 1.37]	0.03	11.91	3	75	0.008	
15cmH_2_O<DP	3	936	1.18 [0.95, 1.46]	0.14	4.06	2	51	0.13	

## Data Availability

All data generated or analyzed during this study are included in this published article. The data used to support the findings of this study are available from the corresponding author upon request.

## Abbreviations

ARDS: Acute respiratory distress syndrome
ICU: Intensive care unit
V_T_: Tidal volume
Pplat: Plateau
PBW: Predicted body weight
DP: Driving pressure
PEEP: Positive end-expiratory pressures
PRISMA: Preferred reporting items for systematic reviews and meta-analyses statement
RCTs: Randomized controlled trials
RR: Relative risk
ROS: Retrospective observational study
ECMO: Extracorporeal membrane oxygenation
POS: Prospective observational study
VILI: Ventilator-induced lung injury
IPD: Individual patient data.

## Supplementary Materials

Supplementary 1 Supplementary Appendix 1. PRISMA Checklist.

Supplementary 2 Supplementary Appendix 2. Search strategy used for the literature review.

Supplementary 3 Supplementary Figure S1. Sensitivity analysis by excluding study by Chiu et al.

Supplementary 4 Supplementary Figure S2. Sensitivity analysis by excluding study by Raymondos et al.
