# Supplementary material for: Higher vs. Lower DP for Ventilated Patients with Acute Respiratory Distress Syndrome: A Systematic Review and Meta-Analysis
Source: Emerg Med Int. 2019 Jul 18;2019:4654705. doi: 10.1155/2019/4654705 (PMC6668539; doi:10.1155/2019/4654705)
Supplement: Supplementary 1 — Supplementary Appendix 1. PRISMA Checklist. [file 4654705.f1.doc]

| **Section/topic** | **#** | **Checklist item** | **Reported on page #** |
| --- | --- | --- | --- |
| **TITLE** | | |  |
| Title | 1 | Higher vs Lower driving pressure for ventilated patients with acute respiratory distress syndrome: a systematic review and meta-analysis | 1 |
| **ABSTRACT** | | |  |
| Structured summary | 2 | Objectives: Driving pressure is recent promising mediator for identification of the effects of mechanical ventilation on outcome in the acute respiratory distress syndrome. The aim of this study was systematically and quantitatively to assess prognostic significance of driving pressure among ventilated patients with acute respiratory distress syndrome  Methods: PubMed, the Cochrane Library, ISI web of knowledge and Embase were systematically searched from inception to June 2018. Two investigators conducted the processes of literature search study selection, data extraction, and quality evaluation independently. RevMan 5.3 software were used for all statistical analyses.  Results: A total of seven studies comprising 8010 patients were included in this meta-analysis. Higher driving pressure had a significant association with higher mortality (pooled risk ratio, 1.10; 95% [CI], 1.05–1.16; *I*^2^ =58%). Sensitivity analysis indicated that one study significantly affected the stability of pooled results. One of the subgroups investigated—ARDS severity—could account for the heterogeneity.  Conclusion: Higher driving pressure were significantly associated with an increased risk of death among ventilated patients with acute respiratory distress syndrome. Future prospective clinical trials is needed to address the unresolved questions about optimum cut-off values of driving pressure and ascertain the benefit of using driving pressure to set volume. | 2 |
| **INTRODUCTION** | | |  |
| Rationale | 3 | Acute respiratory distress syndrome (ARDS) is a common disease that affects up to 46% of mechanically ventilated patients during an intensive care unit (ICU) stay. In spite of decades of research, Seldom effective therapeutic strategies for treating clinical ARDS have appeared. Current treatments focus on support, and the preferred effective therapies, namely lung protective ventilation, act by limiting the iatrogenic injury that is linked to mechanical ventilation. Lung protective ventilation, which is a cornerstone life-saving treatment for acute respiratory distress syndrome (ARDS), includes several components, the most important of which is lowering tidal volume (VT) ,limiting plateau (Pplat) to or below 30 cm H2O and higher positive end-expiratory pressures (PEEPs). This combined strategy is indeed the valid ventilator intervention that has been indicated to prominently improve survival thus far. | 3 |
| Objectives | 4 | a retrospective analysis of several trials in patients with ARDS comparing different PEEP levels at the same VT or different VT levels at the same PEEP, or a combination of both, found that DP was the variable most strongly related to mortality compared with Pplat.11 Nevertheless, because it was a single study, additional data were needed to more clearly understand the potential value of DP for ARDS treatment. We executed a systematic review and meta-analysis to add further documentation that the driving pressure of the tidal cycle is a key factor in the prediction of mortality in mechanically ventilated patients with ARDS to provide reliable evidence to assist physicians in making clinical decisions | 3 |
| **METHODS** | | |  |
| Protocol and registration | 5 | The present meta-analysis was reported according to the Preferred Reporting Items for Systematic Reviews and Meta-analyses Statement (PRISMA). | 4 |
| Eligibility criteria | 6 | Randomized controlled trials (RCTs), controlled studies, cohort studies, and case-control studies were considered eligible if they prospectively collected data on mortality in ventilated adult patients with acute respiratory distress syndrome with driving pressure (DP) measurement. The exclusion criteria were as follows: editorials, reviews, abstracts or conference proceeding, expert opinions, animal experiments, unrelated intervention or outcomes, and insufficient information to extract data. | 4 |
| Information sources | 7 | Four databases, including PubMed, the Cochrane Library, ISI Web of Knowledge and Embase from inception to January 2018. | 4 |
| Search | 8 | Four databases, including PubMed, the Cochrane Library, ISI Web of Knowledge and Embase from inception to August 2018.The following terms were searched in [Title/Abstract]: (“driving pressure”) AND (“acute respiratory distress syndrome” OR “ARDS”). No language restriction was applied for article selection. Additional studies were identified by reviewing the reference lists of relevant articles. | 4 |
| Study selection | 9 | Two authors independently screened title and /or abstract produced by the search. Full-text manuscripts that were identified as relevant were obtained and then assessed independently against inclusion and exclusion criteria. Discrepancies were settled by discussion and/or by introducing other reviewers. | 6 |
| Data collection process | 10 | Two investigators respectively extracted the following descriptive data from all eligible studies, including the authors, year of study, country of origin, study design, study settings, relevant population, sample size, mean age, the optimal cutoff values, outcome assessment, and follow-up period. The primary endpoints of this analysis were mortality outcomes and higher driving pressure. Additionally, a study by Villar et al.Contains two data sets (derivation cohort and validation cohort). | 4 |
| Data items | 11 | the authors, year of study, country of origin, study design, study settings, relevant population, sample size, mean age, the optimal cutoff values, outcome assessment, and follow-up period. | 4 |
| Risk of bias in individual studies | 12 | Newcastle Ottawa Scale for cohort studies was used to assess the reporting quality of the included component studies. This scale comprises eight items evaluating the quality of observational cohort studies in terms of selection, comparability, and outcome. The methodological quality of RCTs was assessed by the Cochrane risk of bias tool.Observational cohort studies and RCTs achieving six or more stars were considered to be of high quality. | 5 |
| Summary measures | 13 | RevMan 5.3 software from Cochrance Collaboration was utilized for the meta-analysis. Relative risk (RR) was reported to estimate the predictive value of higher driving pressure on mortality rate. RR and its relevant 95% CI were pooled by using fixed-effect or random-effect models (the DerSimonian and Laird method) . RR more than 1 indicated beneficial effect of the exposure for mortality | 5 |
| Synthesis of results | 14 | Relative risk (RR) was reported to estimate the predictive value of higher driving pressure on mortality rate. RR and its relevant 95% CI were pooled by using fixed-effect or random-effect models (the DerSimonian and Laird method) . RR more than 1 indicated beneficial effect of the exposure for mortality. Heterogeneity was evaluated by using Cochrane’s Chi^2^ test and I^2^ test. The random-effects model was used if there was heterogeneity between studies | 5 |

Page 1 of 2

| Section/topic | # | Checklist item | Reported on page # |
| --- | --- | --- | --- |
| Risk of bias across studies | 15 | Funnel plots were used to screen for potential publication bias | 5 |
| Additional analyses | 16 | In addition, to investigate the potential sources of heterogeneity in the eligible studies, sensitivity and subgroup analyses were conducted to determine the sources of variability among potentially influencing factors. | 5 |
| RESULTS | | |  |
| Study selection | 17 | The initial search identified 89 citations from PubMed, 153 from the Cochrane Library, 300 from the ISI Web of Knowledge, and 136 from Embase. After removing 325 duplicates, the titles and abstracts of the remaining 353 papers were screened. After 326 records were eliminated by inspection of the titles and abstracts, 26 articles were subsequently scrutinized by a reading of the full text. | 6 |
| Study characteristics | 18 | There is just only one randomized controlled trials (RCTs), three retrospective observational studies and four prospective observational studies, all of which were published between 2015 and 2017. With respect to the clinical setting, all eight studies were conducted in the intensive care unit (ICU). The sample sizes varied across the studies, ranging from 150 to 3562, and the mean age of the patients was between 50.3 and 62.8 years. In terms of population, four studies focused on mild to severe ARDS patients, and four studies focused on moderate to severe ARDS patients. In regard to criteria for selecting the DP thresholds, each studies provided optimum cutoff points. The cutoff values of DP for the prediction varied across the studies, ranging from 13 to 21 cmH2O, with the exception of the study by Raymondos et al. where the cutoff point was not reported. | 6 |
| Risk of bias within studies | 19 | The results showed that three studies scored 9 points, and the remaining studies scored 8 points. That is to say, there is a low risk of bias for the included studies. | 6 |
| Results of individual studies | 20 | In a meta-analysis of eight studies containing 8010 patients, higher DP was significantly associated with increased mortality among mechanically ventilated ARDS patients (pooled risk ratio, 1.10; 95% [CI], 1.05–1.16; I2 =58%). | 6 |
| Synthesis of results | 21 | In a meta-analysis of eight studies containing 8010 patients, higher DP was significantly associated with increased mortality among mechanically ventilated ARDS patients (pooled risk ratio, 1.10; 95% [CI], 1.05–1.16; I2 =58%). | 6 |
| Risk of bias across studies | 22 | Publication bias was considered to be no obvious | 7 |
| Additional analysis | 23 | Considering the remarkable heterogeneity observed, a sensitivity analysis was performed to explore the heterogeneity. After omitting one study Laffey by et al, the heterogeneity of the pooled RR (1.08; 95% [CI], 1.04–1.12; I^2^ =40%) relatively decreased from moderate to low degree, with the I^2^ index decreasing from 58% to 40%. Additionally, subgroup analyses were performed based on ARDS severity, sample size and cutoff values. One of the subgroups investigated—ARDS severity—could account for the heterogeneity. In mild to severe ARDS subgroup, the pooled RR was 1.19(95% CI, 1.07–1.33; I^2^ =47%).In mild to severe ARDS subgroup, the pooled RR was 1.06(95% CI, 1.02–1.09; I^2^ =20%). | 6-7 |
| **DISCUSSION** | | |  |
| Summary of evidence | 24 | The present systematic review and meta-analysis investigated the prognostic significance of DP among ventilated patients with acute respiratory distress syndrome. Accordingly, the pooled risk ratio is 1.10 (95% CI, 1.05–1.16), indicating that higher DP is a bedside available parameter for the prediction of mortality in ventilated patients with acute respiratory distress syndrome that may help identify patients prone to develop VILI and at increased risk of death. | 8 |
| Limitations | 25 | Several limitations of this study should be discussed. First, First, marked heterogeneity existed across the included studies in terms of the ARDS severity, sample size and optimal cutoff values. Although we performed sensitivity and subgroup analyses to explore the sources of potential heterogeneity between studies, the heterogeneity of each parameter was not entirely decreased. Additional high-quality studies with a broader spectrum of clinical settings may be required. Second, the cutoff points of DP varied, ranging from 13 to 21 cmH2O, we could not determine ideal cutoff values for DP because we did not have the initial data to construct ROC curves. To confirm whether one or more DP thresholds exist, further explorations in larger, prespecified groups of patients are required. Finally, As to whether data rooted in each trial, namely numerical value of driving pressure, are contaminated by several confounding factors that strongly influence (e.g., spontaneous effort, chest wall stiffness and position) or not, we did not know. Simultaneously, virtually none of the trials assured that driving pressure was recorded under passive conditions, since plateau pressure can be displayed by most ventilators even when the patient is actively breathing. Again, chest wall stiffness and in some cases position influence those numbers as well. Driving pressure will change in the same patient as disease and ventilation settings do. Therefore, the accuracy of DP needs to be further studied to evaluate the value. | 9 |
| Conclusions | 26 | Higher DP was significantly associated with an increased risk of death among ventilated patients with acute respiratory distress syndrome, and its prognostic performance is convenient for clinical utility. Future prospective clinical trials is needed to address unresolved questions about optimum DP cut-off values and ascertain the benefit of using DP to set volume. | 10 |
| **FUNDING** | | |  |
| Funding | 27 | Not application | 10 |
