# Supplementary material for: Higher vs. Lower DP for Ventilated Patients with Acute Respiratory Distress Syndrome: A Systematic Review and Meta-Analysis
Source: Emerg Med Int. 2019 Jul 18;2019:4654705. doi: 10.1155/2019/4654705 (PMC6668539; doi:10.1155/2019/4654705)
Supplement: Supplementary 2 — Supplementary Appendix 2. Search strategy used for the literature review. [file 4654705.f2.docx]

Supplementary Appendix. Search strategy

Search strategy used for the literature review. These databases were searched up to June 21, 2018.

| PubMed |  |  |
| --- | --- | --- |
| Search #1 | (“driving pressure*”[Mesh]OR driving pressure* [tiab]OR Pressure*”[Mesh]OR pressure* [tiab]) | 757599 |
| Search #2 | (“acute respiratory distress syndrome”[Mesh]OR acute respiratory distress syndrome [tiab]OR ARDS[tiab]) | 15895 |
| Search #3 | #1 AND #2 | 3266 |
| the Cochrane Library |  |  |
| Search #1 | (driving pressure* OR pressure* ):ti,ab,kw | 106120 |
| Search #2 | (“acute respiratory distress syndrome OR ARDS) :ti,ab,kw | 1963 |
| Search #3 | #1 AND #2 | 678 |
| ISI Web of Knowledge |  |  |
| Search #1 | “driving pressure*”[Mesh] | 5782624 |
| Search #2 | “acute respiratory distress syndrome”[Mesh] | 43960 |
| Search #3 | #1 AND #2 | 9985 |
| Embase |  |  |
| Search #1 | ‘driving pressure*’/exp OR ‘driving pressure*:ab,ti OR pressure*:ab,ti | 1023672 |
| Search #2 | ‘acute respiratory distress syndrome’/exp OR ‘acute respiratory distress syndrome :ab,ti OR ARDS:ab,ti | 16456 |
| Search #3 | #1 AND #2 | 4567 |
